# Solitary Plasmacytoma in the Calcaneus

**DOI:** 10.7759/cureus.37637

**Published:** 2023-04-16

**Authors:** Maram Albandak, Aya Mikkawi, Laith A Ayasa, Yousef Ansara, Mohammed Janajri

**Affiliations:** 1 Internal Medicine, Al-Quds University, Jerusalem, PSE; 2 Internal Medicine, An-Najah National University, Nablus, PSE

**Keywords:** plasma cell dyscrasias, calcaneus, plasma cell tumor, plasmacytoma, trauma

## Abstract

Solitary plasmacytomas (SPs) are tumors characterized by local monoclonal plasma cell proliferation, presenting without systemic manifestations. It mainly affects the axial skeleton, with calcaneal involvement being extremely rare. We report a case of a 48-year-old patient with a history of gunshot injury to his foot who presented with worsening heel pain and a calcaneal cyst. Biopsy revealed plasmacytoma, and subsequent 18F-fluorodeoxyglucose positron emission tomography/computed tomography (18F-FDG PET/CT) scan further supported the diagnosis of solitary plasmacytoma of the bone (SPB). Management included lesion excision, bone cement placement, and radiotherapy. However, due to recurrent osteomyelitis following cement placement, the patient eventually required total calcanectomy. SPB usually affects older adults, and developing the disease at a young age and in the calcaneus is exceedingly uncommon. Trauma is implicated as a possible inciting trigger in the pathogenesis of SPB without a clear association. This case highlights the importance of developing our current understanding of the clinical presentation and manifestations of SPB, beyond the conventional assumption that it only affects the axial skeleton of older individuals.

## Introduction

Plasmacytomas are tumors characterized by the proliferation of monoclonal plasma cells in soft tissue or bone, presenting without systemic manifestations [1]. Based on the number of lesions, they are subdivided into solitary plasmacytoma (SP) and multiple solitary plasmacytoma [1]. SP is further categorized based on the location of the tumor into SP of the bone (SPB) and extramedullary plasmacytoma (EMP) [2]. SPB is more common, accounting for approximately 70% of SPs, and has a male predominance with a median age at diagnosis ranging from 55-60 years [3]. Signs and symptoms of the disease include a painful single bone lesion, swelling of the bone, or an incidentally discovered bone lesion on routine radiological studies [4]. SPB typically involves the bones of the axial skeleton, such as the skull and vertebrae, with only a few cases involving the appendicular skeleton and an even smaller percentage limited to the bones of the hands and feet [5]. This report describes a unique case of SPB in the calcaneus, a very rare site of the disease, following a remote history of trauma with distinct clinical features.

## Case presentation

We report a case of a 48-year-old male with a past medical history significant for a gunshot injury to his right foot, hypertension, and poorly controlled diabetes mellitus. His right hindfoot was injured in 1986, after which the patient started complaining of recurrent foot pain at the same site. This pain was notably exacerbated by cold temperatures and was alleviated by over-the-counter analgesic medications.

**Figure 1:**
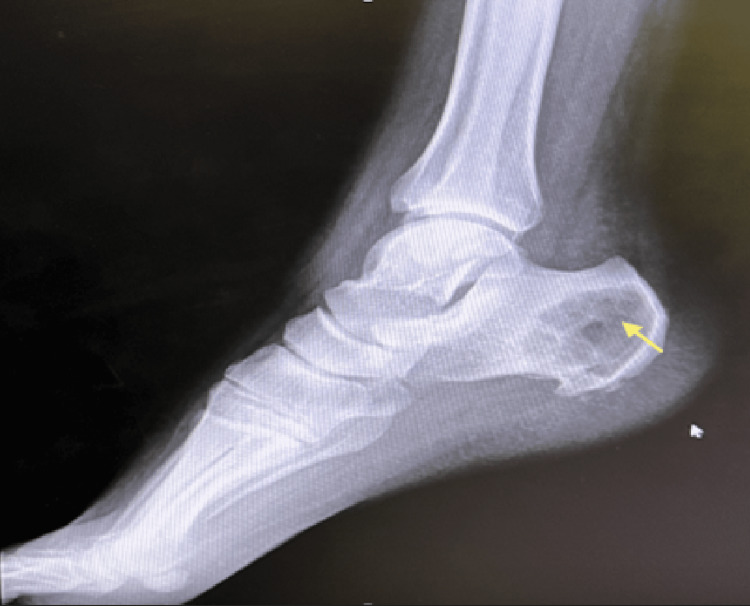
Initial X-ray of the right foot X-ray of the right foot showing a 4.3x3.5 cm lytic lesion with a soap bubble appearance (yellow arrow) at the posterior part of the right calcaneus with a wide transition zone.

**Figure 2:**
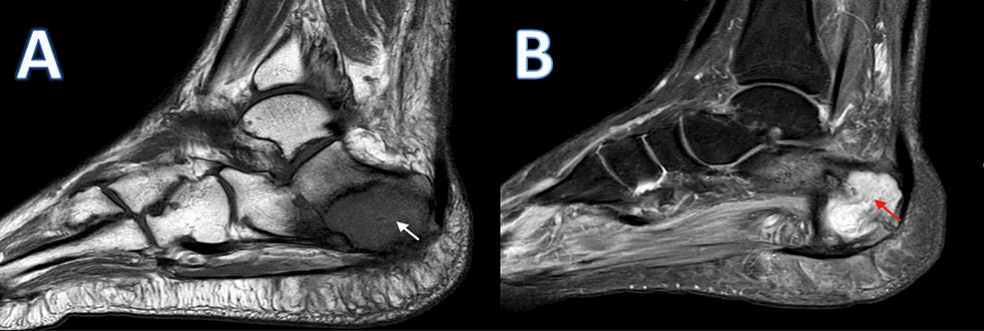
T1-weighted and T2-weighted MRI of the right foot (A) Right hypointense calcaneal marrow infiltrative lesion measuring 4.6x3 cm on T1 (white arrow) and (B) isointense to hyperintense on T2 (red arrow). The lesion demonstrates vivid enhancement after contrast administration, along with bone expansion and cortical breaching.

**Figure 3:**
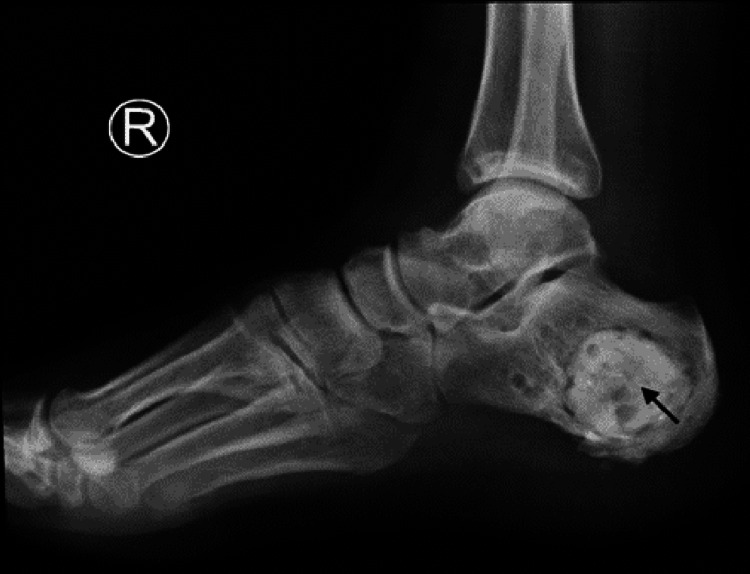
X-ray of the right foot demonstrating bone cement in the right calcaneus (black arrow)

In 2020, the patient sought medical attention due to increasing pain intensity associated with a 15-kilogram weight loss over the past nine months. An X-ray revealed a lytic lesion at the posterior part of the right calcaneus (Figure 1). Initially, a calcaneal abscess was suspected and treated with incision and drainage, resulting in temporary relief of symptoms. Two months later, the patient still experienced persistent ankle pain, prompting further workup. The patient's laboratory values disclosed no abnormally elevated proteins, or anemia. Calcium levels and kidney function were normal, as summarized in Table 1. Magnetic resonance imaging (MRI) revealed a right calcaneal bone cyst (Figure 2). A biopsy of the lesion showed infiltration of monoclonal plasma cells, confirming plasmacytoma. Additionally, 18F-fluorodeoxyglucose positron emission tomography/computed tomography (18F-FDG PET/CT) whole-body scan ruled out the presence of other lesions. Bone marrow (BM) aspiration from the posterior iliac crest revealed normal megakaryocytes with unremarkable morphology. The CD138 (syndecan-1) immunohistochemical staining highlighted a normal number and distribution of plasma cells with an overall proportion of 2-3%, thus ruling out multiple myeloma and confirming the diagnosis of SPB. As a result, calcaneal cyst excision with bone cement placement was performed for reconstruction (Figure 3). The patient also received a total radiation dose of 50 Gray (Gy) with a fractionation size of 2 Gy per day, delivered over a period of 25 treatment days (five days per week), for a total treatment duration of five weeks.

**Table 1:** The patient’s laboratory test values at the time of presentation

Parameter	Test result	Normal range
Hemoglobin (HGB)	16.3 g/dl	13.5-17 g/dl
White blood cell count (WBC)	6.8x10^3/μL	4.6-11* 10^3/μL
Plateletcrit	0.2%	0.22-0.24%
Erythrocyte sedimentation rate (ESR)	10 mm/hr	0-10 mm/hr
C-reactive protein (CRP)	29.5 mg/dl	0-5 mg/dl
Creatinine	0.86 mg/dl	0.7-1.2 mg/dl
Blood urea nitrogen (BUN)	9.6 mg/dl	6-20 mg/dl
Total protein	7.27 g/dl	6.6-8.7 g/dl
B2 microglobulin	1.8 mg/l	1.8-2.2 mg/l
Calcium	10.04 mg/dl	8.6-10 mg/dl
Immunoglobulin A (IgA)	213 mg/dl	90-450 mg/dl
Immunoglobulin G (IgG)	819 mg/dl	800-1800 mg/dl
Immunoglobulin M (IgM)	29 mg/dl	60-280 mg/dl
Kappa	196 mg/dl	138-375 mg/dl
Lambda	100 mg/dl	93-242 mg/dl
Hepatitis B virus antigen (HBV)	Non-reactive	
Hepatitis C virus antibody (HCV)	Non-reactive	

Following cement placement, the patient continued to experience persistent ankle pain due to recurrent attacks of osteomyelitis. As a result, the 18F-FDG PET/CT whole-body scan was repeated. It revealed a focal lucent bone area measuring approximately 4x4x3 cm with internal cement and an associated hypermetabolic sinus tract with an underlying calcaneal bone defect, suggesting chronic osteomyelitis with sinus tract formation. Therefore, the bone cement was removed to eliminate the source of infection and improve the effectiveness of antibiotic therapy.

**Figure 4:**
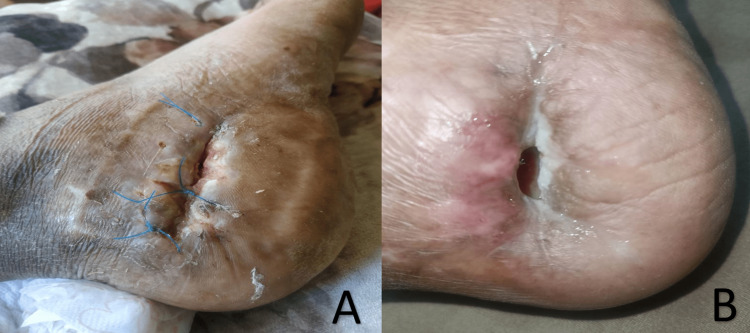
Large skin defect with sinus tract over the right calcaneus due to recurrent osteomyelitis

Despite bone cement removal, the pain lingered as the patient developed persistent osteomyelitis in the calcaneus, necessitating multiple hospitalizations and antibiotic administration. This suggests the possibility that radiation therapy has damaged the irradiated bone tissue, contributing to the patient's persistent osteomyelitis. During his most recent hospitalization, bone cultures grew *Klebsiella pneumoniae*, which was only sensitive to colistin and tigecycline. A new 18F-FDG PET/CT scan showed the persistence of the same lucent bone area with a new medial cortical defect containing fluid and gas densities connected to a lateral skin defect (Figure 4).

**Figure 5:**
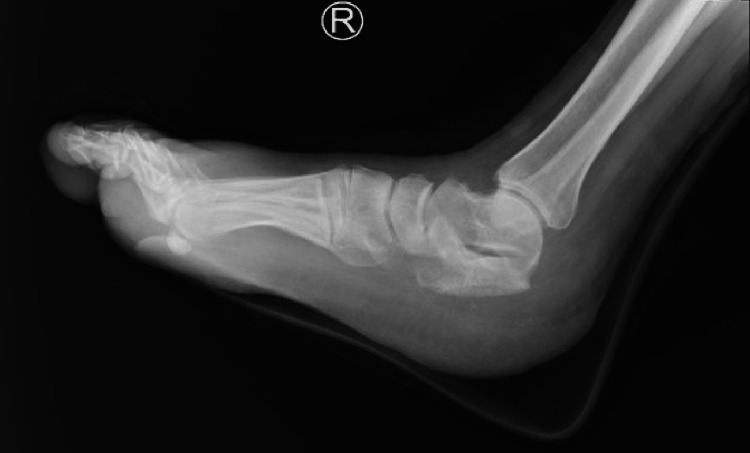
X-ray of the right foot following total calcanectomy

Consequently, due to the recurrent infections and based on his latest MRI findings, a total calcanectomy was planned and performed in February 2023 (Figure 5). The patient was doing well at one month post-operative, with markedly decreased foot pain and a well-healed surgical wound. He gradually regained the use of his right foot and was scheduled to receive physiotherapy over the course of the next few weeks.

## Discussion

**Figure 6:**
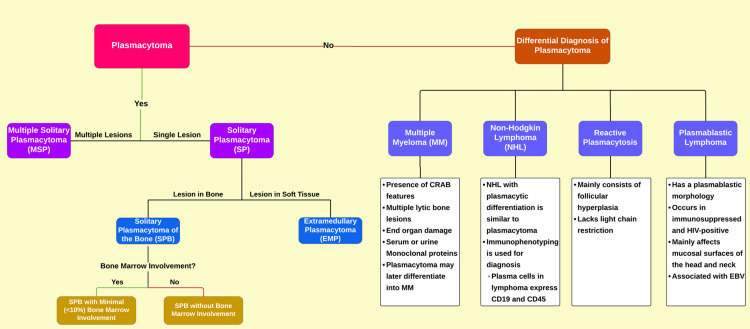
Classification and differential diagnosis of plasmacytoma Information source: Iqbal and Majid, 2022 [1] CRAB: calcium (hypercalcemia), renal insufficiency, anemia, or bone lesions; EBV: Epstein–Barr virus

Plasmacytoma is an uncommon, early-stage, plasma cell dyscrasia that usually affects bones of the axial skeleton, such as the skull, vertebrae, ribs, sternum, and pelvis, as well as proximal long bones of the hand and feet. It has also been described in mucosal surfaces [6]. It is considered to be an intermediate phase between monoclonal gammopathy of undetermined significance (MGUS) and multiple myeloma. SP is the most common form of plasmacytoma. It is further divided into SPB arising from plasma cells of the bone marrow, and EMP, arising from plasma cells of mucosal surfaces (Figure 6) [1,7]. The frequency of SPB is 40% higher than that of EMP [8].

SP comprises 2-5% of all plasma cell cases [8]. The presence of a single biopsy-proven bony or extramedullary lesion defines SP, particularly in the setting of a normal BM examination without clonal plasma cells. Moreover, it requires the absence of radiological evidence on MRI or CT of skeletal lesions (except for the primary solitary lesion), normal skeletal survey, and the absence of end-organ involvement, such as the CRAB (calcium (hypercalcemia), renal insufficiency, anemia, bone lesions) manifestations of multiple myeloma [7].

The exact etiology of SPB remains unknown; however, multiple hypotheses have implicated the role of viral infections, radiation, chemical inhalation, and genetic factors as probable factors. It is a disease of older adults, with a median age of 55 years; thus, its development in young adults is extremely rare [6]. The association between SPB at a young age and a history of preceding trauma has been elucidated in prior studies. However, the exact significance of this association is yet to be established [6,9,10]. The hallmark of plasma cell development involves antigen stimulation of mature naive B cells, prompting further proliferation, heavy chain class switching, and differentiation into memory B cells or plasma cells [10]. Trauma can lead to enhanced cytokine release, contributing to plasma and stromal cfvell proliferation in the bone, possibly leading to plasmacytoma development. Dysregulated interleukin-6 (IL-6) production and accompanying MYC oncogene expression were found to cause more rapid plasmacytoma development in a mouse model study conducted by Rutsch et al. This was hypothesized to establish a “feed-forward cytokine amplification loop,” which creates an optimal environment for plasmacytoma growth and progression [6,10].

SP is more common in males with a male-to-female ratio ranging from 1.2:1 to 2:1. SPB more frequently affects the bones of the axial skeleton such as the vertebrae, whereas EMP more frequently affects the regions of the head and neck, with the two entities differing in clinical course and prognosis. Patients with SPB usually seek medical attention due to pain from bone involvement and spinal cord or nerve root compression [8]. Interestingly, Kissel et al. described a case of SP in the talus presenting as a pathologic fracture in a 77-year-old male with a previous history of trauma [11]. Our patient presented with worsening chronic discomfort in his heel, years after the initial trauma. Hence, we speculate that the trauma may have played a role in the pathogenesis of his lesion by acting as a stimulus for subsequent plasma cell proliferation and clonal neoplasm formation.

The bones of the hand and foot are extremely unusual locations for plasmacytoma development, even for primary bone tumors. The differential diagnosis comprises both primary bone lesions such as osteogenic sarcoma, chondrosarcoma, fibrosarcoma, liposarcoma, and round cell tumors, in addition to rare metastatic foot lesions from the breast, lung, colon, and renal malignancies. Osteomyelitis is another potential diagnosis for lesions that present with calcaneal pain, swelling, and tenderness. However, the presence of puncture wounds, constitutional symptoms such as fever, and local signs of inflammation can help distinguish between the two [6].

The International Myeloma Working Group (IMWG) has established criteria for diagnosing SPB. The criteria include the following: (1) normal skeletal survey and the absence of any other lytic lesions, (2) biopsy-proven solitary tumor of bone with evidence of clonal plasma cells, (3) the presence of plasma cells within the biopsy, and (4) the absence of clonal plasma cells in the BM aspirate. In order to diagnose SPB, other conditions, such as multiple myeloma, must be ruled out by examining the BM, along with ensuring the absence of CRAB manifestations described above [12]. Using CT with PET scan is considered the gold standard for identifying SPB lesions [13]. MRI is useful in tumor staging, where SPB appears as hypo-/iso-intense on T1-weighted images and hyperintense on T2- weighted images, as evident in our case [14]. It is also helpful in the verification of tumor reduction after therapy.

It is reported that roughly 50% of patients diagnosed with SPB are at risk of developing MM, with the median time of progression being two to three years [15]. Given its relevant prognostic value, it is considered clinically significant to determine the degree of plasma cell infiltration in the BM [16]. Hill et al. have documented this correlation in their study, where 68% of the cases that progressed to MM displayed a clonally related plasma cell population in the BM [17]. Other prognostic values related to poor prognosis and progression to MM include age >40 years, tumor size >5 cm, and the degree of angiogenesis [18].

With a control rate of approximately 80%, localized radiotherapy is considered the treatment of choice for SPB [13]. There is no established optimum radiation dosage for SPB, and the recommended dose ranges vary across different guidelines. Radiation therapy doses ranging from 30-60 Gy were used over four weeks, with the majority of studies advocating the use of 35-50 Gy [19]. Surgery and systemic chemotherapy are two other debatable options for managing SPB. Generally, surgery is indicated in cases of fractures, vertebral instability, or for neural decompression, mandating a multidisciplinary approach for decision-making, including neurological and orthopedic evaluation [16]. As for systemic chemotherapy, most studies have not reported an additional beneficial effect on disease progression or complication prevention [2]. However, it has been shown to slow the progression of multiple myeloma without affecting its incidence [13]. Consistent with previous findings, Ozsahin et al. conducted a retrospective analysis of 206 patients with SPB and 52 patients with EMP, who were treated by either radiotherapy alone (214 patients), radiotherapy in combination with chemotherapy (34 patients), or surgery alone (eight patients). Patients who underwent localized radiation therapy had a lower incidence of local recurrence compared to those who did not receive radiation (12% versus 60%) [19].

Patients diagnosed with SPB typically have a median overall survival of approximately 10 years. 50-60% of these patients progress to develop multiple myeloma, which is possibly explained by the persistence of undetectable tumor cells not lying within the field of radiotherapy [20]. These findings reiterate the significance of regular follow-up after completion of the radiation therapy course. Patients are advised to undergo regular laboratory testing, including urine and serum protein electrophoresis with immunofixation, complete blood count, serum creatinine, and serum calcium every four to six months during the first year and annually after that [13].

## Conclusions

We have described the case of a middle-aged man who presented with SPB in a relatively rare location, most likely due to trauma endured years prior. SP can infrequently arise in the appendicular skeleton of young patients, particularly in those with a history of trauma. The exact significance of the association between SPB at a young age and a history of preceding trauma remains to be established. A biopsy of the lesion with evidence of clonal plasma cells is required for establishing the diagnosis. Moreover, it is fundamental to exclude multiple myeloma before initiating treatment. Localized radiotherapy remains the best treatment modality for SP, and regular follow-up is essential for monitoring disease progression to multiple myeloma and addressing disease recurrence or delayed complications.
